# Weaving a new web: gregarious parasitism in *Idris* Förster (Hymenoptera: Scelionidae) attacking spider eggs

**DOI:** 10.1371/journal.pone.0319209

**Published:** 2025-02-25

**Authors:** K. Rajmohana, Rupam Debnath, V. Sushama, Souvik Sen, K. P. Dinesh

**Affiliations:** 1 Zoological Survey of India, Kolkata, West Bengal, India; 2 Department of Zoology, University of Calcutta, Kolkata, West Bengal, India; 3 Zoological Survey of India, Pune, Maharashtra, India; Chulalongkorn University, THAILAND

## Abstract

The present study, conducted in West Bengal, India, explored the unique ‘multi-chambered’ appearance of certain spider eggs, leading to the discovery of gregarious parasitism in the idiobiont endoparasitoid *Idris* Förster (Hymenoptera: Scelionidae). Little is known about the roles of parasitoid Hymenoptera in regulating spider populations. Phylogenetic analysis based on the mitochondrial cytochrome c oxidase I marker identified five distinct species of *Idris*. These five gregarious species, identified in association with various spider hosts across multiple locations during a two-year study, offer new insights into host-parasitoid interactions and their adaptability in different host systems. Additionally, six novel host associations between egg parasitoids and five spider species from two families are documented. Under the family Scelionidae, *Idris* is the second genus, after *Telenomus* Haliday, adapting to gregarious development. Our findings emphasize the existence of diverse trophic interactions and life strategies in nature that are yet to be documented.

## 1. Introduction

Parasitoid Hymenoptera is recognized as key natural enemies of arthropods [1], ubiquitous in multitrophic interactions and ecological communities [2]. Although they mainly target insects, some of them also attack the eggs or the developing stages of non-insect groups like spiders and mites [3,4]. Given the significance of spiders in mediating trophic cascades as important predators and biological control of insects in natural communities, their regulation, in turn, by parasitoids gains relevance [5]. However, detailed understanding of the biology, development, and behaviour of spider egg parasitoids is limited [6,7].

Parasitoids display different developmental strategies like solitary or gregarious based on their brood production and larval development [8,9]. Most parasitoid wasps are solitary, allowing only one offspring to develop on or within a single host [10]. When more than one offspring completes their development on or within a host without siblicide, they are classified as gregarious [10]. Thus far, all known endoparasitoids of spider eggs are solitary species [11].

Members of the tribe Baeini (Scelionidae) are one of the major biotic sources of mortality of spiders [4,12]. They have been reared from 20 families of araneomorph spiders to date [4,12,13]. *Idris* Förster is cosmopolitan [14] and the most speciose genus within the tribe Baeini [12]. To date, 173 species of *Idris* have been described globally, including 41 from India [7,12,15–17]. *Idris* has a ground plan biology as solitary primary idiobiont endoparasitoids of spider eggs [11,18]. In a single documented instance, *Idris elba* Talamas was reported parasitizing the eggs of *Bagrada hilaris* (Burmeister) (Hemiptera: Pentatomidae) [15]. This can even be an opportunistic behaviour of *Idris*, demonstrating their ability and adaptability to parasitize and successfully develop on hosts potentially unrelated to its typical host range.

During our studies on egg parasitoids of spiders [7,19], we encountered a few ‘multi-chambered’ spider eggs, and multiple progeny emergence of *Idris*, both of which previously unknown in science and representing a case of gregarious parasitism. Traits of the parasitoid, including their sex ratio were explored. Using mitochondrial cytochrome c oxidase I (mt COI), the genetic distinctness of all five recorded species of *Idris* was confirmed. Additionally, novel host associations between these wasps and five spider species, spanning two spider families, were documented.

## 2. Materials and methods

### 2.1. Field sampling and rearing

This study was conducted from February 2021 to December 2023 in West Bengal, India. Approximately 800 spider egg sacs were systematically handpicked from agroecosystems and surrounding natural habitats. Collections were made from different locations (Table 1), various habitats, including leaf litter, low vegetation, and tree trunks. Mother spiders guarding the eggs, if present, were also collected. The spider egg sacs were placed in labelled vials, while the mother spiders were preserved directly in absolute alcohol. The egg sacs were brought to the laboratory and reared at room temperature, 24–25°C. The emergence of both spiderlings and parasitoids was recorded (Table 1). Emerged individuals were subsequently transferred to absolute alcohol for further morphological and molecular studies. The spider egg sacs were monitored for up to one month from the date of collection, after which they were dissected to document unhatched eggs or unemerged parasitoids.

**Table 1:** Collecting events and host-parasitoid emergence.

Collecting events				Hosts			Parasitoids		
Case	Instance no.	Collection locality	Date of collection	Number of ‘multi-chambered’ eggs in egg sac	Host spider	Number of spiderlings emerged	Parasitoid	Parasitoid emergence after collection of host eggs	Sex ratio of lab emerged parasitoids (m:f)
A	1	Raidighi, South 24 Parganas	21-11-2021	23	*Bianor angulosus* (Karsch)	2	*Idris* sp. 1	7 days	1:40
	2	Kalna, East Burdwan	10-10-2022	33	*Bianor angulosus* (Karsch)	26	*Idris* sp. 1	13 days	7:89
	3	Kalna, East Burdwan	10-10-2022	29	*Bianor angulosus* (Karsch)	8	*Idris* sp. 1	7 days	4:78
	4	Ekghoria, Murshidabad	24-10-2022	26	*Bianor angulosus* (Karsch)	12	*Idris* sp. 1	7 days	9:43
	5	Patharpratima, South 24 Parganas	26-11-2022	24	Neither mother spider nor spiderling could be documented	–	*Idris* sp. 1	2 days	0:9
	6	Kakdwip, South 24 Parganas	09-11-2023	14	*Bianor albobimaculatus* (Lucas)	13	*Idris* sp. 1	3 days	3:54
B	7	Raidighi, South 24 Parganas	28-11-2022	42	*Neoscona* sp.	75	*Idris* sp. 2	8 days	152:0
							*Odontacolus markadicus* Veenakumari	8 days	4:65
C	8	Raidighi, South 24 Parganas	28-11-2022	14	Neither mother spider nor spiderling could be documented	–	*Idris* sp. 3	4 days	0:9
D	9	Hansqua, Darjeeling	14-12-2022	26	*Hyllus semicupreus* (Simon)	2	*Idris* sp. 4	10 days	2:26
E	10	Nobanda, Bankura	02-11-2023	10	*Harmochirus brachiatus* (Thorell)	1	*Idris* sp. 5	3 days	2:30

### 2.2. Morphological studies

The taxonomic identification of the parasitoids followed Masner [19] for generic identification and Valerio et al. [20] for species level identification of *Odontacolus* Kieffer, while for the identification of spiders Tikader [21], Barrion & Litsinger [22] and Prószyński [23] were consulted. Since the updated key is not available for *Idris*, morphospecies were segregated based on the diagnostic characters outlined for species description by Johnson et al. [12] and Debnath et al. [19]. For morphological studies and digital imaging, Leica M205A stereomicroscope with 1 × objective fitted with a Leica DFC 500 digital camera was used. The images were later processed using LAS version 3.6 extended focus software. The voucher specimens are deposited in the National Zoological Collections (NZC) of Zoological Survey of India (ZSI), Kolkata.

### 2.3. Molecular studies

While the mother spiders were identified based on their morphology, the spiderlings were identified through DNA barcoding. Genomic DNA was extracted from the spiderlings and *Idris* spp. using the DNeasy Blood and Tissue Kit (QIAGEN, Inc.) according to the kit protocol (destructive method). DNA quantitation was performed using a Qubit 2.0 fluorometer, and polymerase chain reaction (PCR) amplification for mt COI was carried out using the primers: LCO1490 and HCO2198 [24]. The PCR reactions followed the protocol described by Debnath et al. [19], with a thermal cycling profile as in Gariepy et al. [25].

Positive PCR products were confirmed on agarose gel via electrophoresis and subsequently purified. The purified products were sequenced bidirectionally using Sanger’s dideoxy method on an ABI 377 sequencer (Applied Biosystems). Chromatogram files were manually assessed for quality, and the sequences were submitted to the National Center for Biotechnology Information (NCBI) GenBank (S1 Table). Pairwise nucleotide sequence distances within and among *Idris* spp. were calculated using the Kimura 2-parameter model (K2P) of substitution, implemented in MEGA X [26].

### 2.4. Molecular phylogeny and species delimitation

A total of 402 mt COI sequences of *Idris* were retrieved from the NCBI GenBank and Barcode of Life Data Systems (BOLD) databases on November 4, 2024. These sequences were aligned with 10 *Idris* sequences generated in this study (S1 Table) using MEGA X [26]. In addition, six sequences of Scelionidae with the highest percentage identity to our *Idris* query sequences from the BLAST search were also included in the analysis (see below). A maximum likelihood (ML) phylogenetic tree was constructed using 412 sequences (S1 Table) in IQ-TREE multicore version 1.6.12 [27] with 1000 ultrafast bootstrap replicates, employing the TIM2 + F + I + G4 substitution model, which was auto-selected based on the Bayesian Information Criterion under default parameters. The consensus tree was visualized using FigTree version 1.4.4 (https://tree.bio.ed.ac.uk/software/figtree/), with *Trissolcus basalis* (Wollaston) designated as the outgroup [12]. Assemble Species by Automatic Partitioning (ASAP) was used for species delimitation [28]. The analysis was performed on the online server (https://bioinfo.mnhn.fr/abi/public/asap/) using Kimura (K80) corrected distances and default parameters. The resulting morphospecies were clustered on the ML tree using FigTree.

The graph was generated using RStudio (https://www.r-project.org/) with packages ‘ggplot2’ [29] and ‘reshape’ [30]. Publicly available digital maps from the Natural Earth database (https://www.naturalearthdata.com/) were obtained in shapefile format and analyzed using the open-source software QGIS version 3.22.12 (http://www.qgis.org). No permits were required for the present study, which complied with all relevant regulations.

## 3. Results

**Fig 1:**
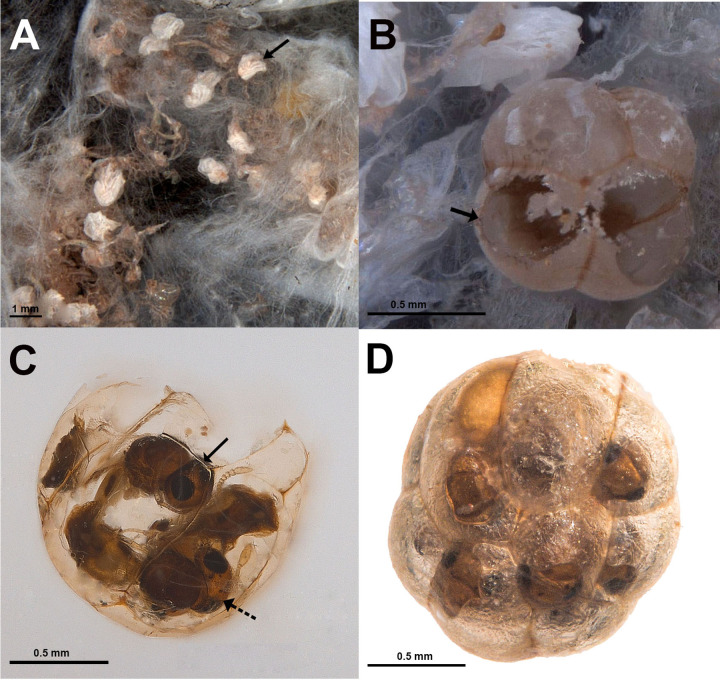
Eggs inside the spider egg sacs. A. Category (i) (indicated with arrow). B. Category (arrow indicates exit hole). C. Category (iii) with both male and female *Idris* sp. 1 (solid arrow = male, dotted arrow = female). D. Category (iv) with 10 ‘chambers’.

Out of the 800 spider egg sacs reared, parasitoid emergence was recorded in only 91 cases, resulting in an overall parasitism success of 11.3%. The eggs inside the spider egg sacs are classified under one of the categories below:

i. host eggs, post-emergence of spiderlings (Fig 1A)
ii. hollow eggs with exit holes, post emergence of solitary/gregarious parasitoids (Fig 1B)
iii. hollow eggs with exit holes but also with some unemerged parasitoids (Fig 1C)
iv. intact eggs without exit holes, containing unemerged parasitoids (Fig 1D)

The eggs in the first category are collapsed, shrivelled, shrunken or broken and could not be counted accurately (Fig 1A). Eggs in the other three categories, whether hosting single or multiple parasitoids, appeared translucent once the parasitoids reached maturity. Eggs hosting multiple parasitoids exhibited a swollen, fused and ‘multi-chambered’ appearance (Fig 1B–1D), with the number of ‘chambers’ corresponding to the number of developing parasitoids. Despite the number of ‘chambers’ or developing parasitoid progenies, these eggs were similar in shape and appearance. Overall, 10 egg sacs contained eggs (n = 241) that appeared ‘multi-chambered’. The meconium was also distinct.

Within an egg sac, all eggs typically had a consistent number of ‘chambers’, except in one case. Among the 10 egg sacs, five contained eggs appearing five ‘chambered’, two were six ‘chambered’, and one had ten ‘chambers’ (Fig 1D). In total, 241 eggs appeared the ‘chambered’ phenotype (26 with 10 ‘chambers’, 83 with 6 ‘chambers’, 76 with 5 ‘chambers’, and 42 with 4 ‘chambers’). Out of the above, fourteen were ruptured; hence, the number of ‘chambers’ could not be ascertained clearly. In total, about 30 eggs contained unemerged parasitoids (categories iii and iv).

**Fig 2:**
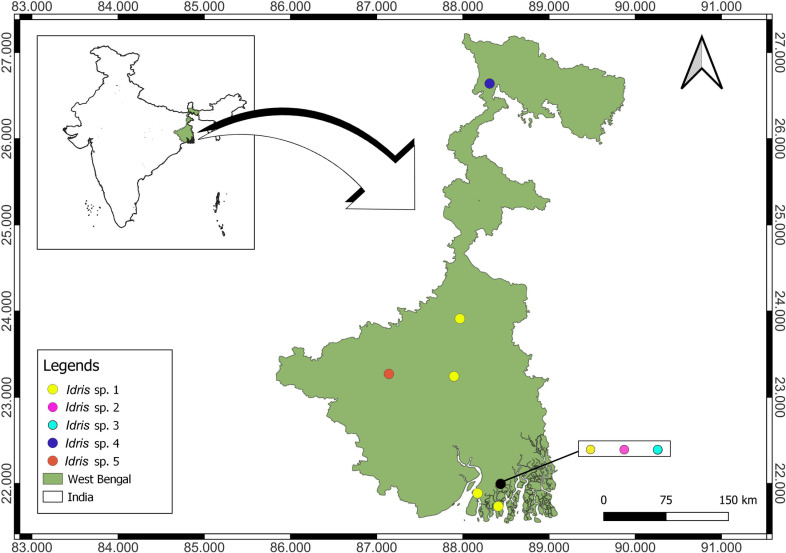
Distribution of gregarious species of *Idris* in this study. Publicly available digital maps from the Natural Earth database (https://www.naturalearthdata.com/) were obtained in shapefile format and analyzed using the open-source software QGIS version 3.22.12 (http://www.qgis.org).

A spatiotemporal analysis of *Idris* species distribution revealed that three species developed gregariously in the same locality (Raidighi). Two are associated with two different spiders while the host of the third could not be documented, as neither the mother spider nor the spiderlings were retrieved (Fig 2, Table 1). There is no parasitoid species overlap recorded in other collection localities (Fig 2).

### 3.1. Taxonomic identification and molecular studies

**Fig 3:**
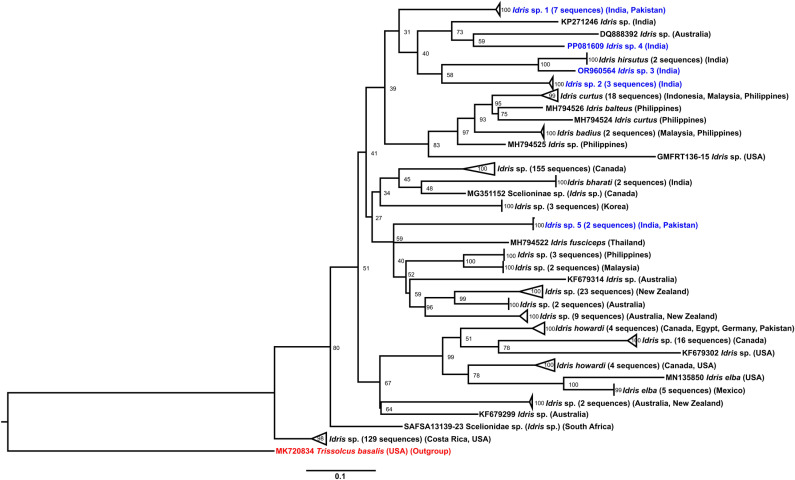
Maximum likelihood tree for *Idris* species based on 647 bp mt COI sequences. The five *Idris* species identified in this study are indicated in blue font colour. Bootstrap values are provided at the nodes, and each lineage is annotated with support from the ASAP analysis.

The ASAP analysis identified 36 species (including the outgroup; S1 Table, S1 Fig), with the phylogenetic analysis (Fig 3) supporting the identification of the *Idris* wasps in this study into five distinct species. Preliminary morphological studies considered taxonomic characters such as proportions and sculpture of metasomal segments, frontal sculpture, wing morphology (including forewing venation), and body colouration, for species delimitation [12,19] and was in agreement with the molecular studies. A detailed species level taxonomic study of *Idris* will be undertaken later. The interspecific genetic distance between the species of *Idris* ranged from 14.1% to 17.6% (Table 2). In one instance, emergence of multiple genera of Baeini such as *Idris* and *Odontacolus* was documented (Table 1). As hosts, five species of spiders from two families were identified: *Neoscona* sp. (Araneae: Araneidae), *Bianor angulosus* (Karsch), *Bianor albobimaculatus* (Lucas), *Hyllus semicupreus* (Simon), and *Harmochirus brachiatus* (Thorell) (Araneae: Salticidae) (Table 1).

**Table 2:** Uncorrected pairwise genetic distance between *Idris* spp. for mt COI (in percentage).

Species	*Idris* sp. 1 (n = 6 sequences)	*Idris* sp. 2	*Idris* sp. 3	*Idris* sp. 4	*Idris* sp. 5
***Idris* sp. 1**	0.0–0.3				
***Idris* sp. 2**	15.7–16.1	0			
***Idris* sp. 3**	14.6–15.0	15.2	0		
***Idris* sp. 4**	15.2–15.5	14.1	14.8	0	
***Idris* sp. 5**	16.4–16.6	16.5	17.6	15.4	0

**Fig 4:**
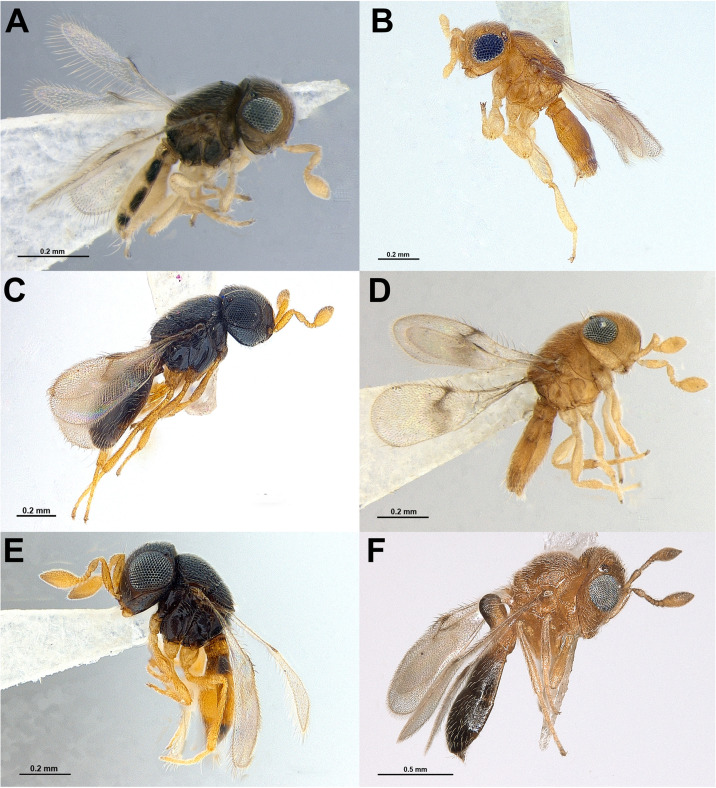
Parasitoids emerged from the spider egg sacs. A. *Idris* sp. 1, ♀; B. *Idris* sp. 2, ♂; C. *Idris* sp. 3, ♀; D. *Idris* sp. 4, ♀; E. *Idris* sp. 5, ♀; F. *Odontacolus markadicus*, ♀.

The BLAST results (as of November 4, 2024) showed a 100% similarity match between *Idris* sp. 1 (Fig 4A) and a Scelionidae sequence from Pakistan (BOLD ID: GMPJA9337-21) (Table 3). *Idris* sp. 2 (Fig 4B) and *Idris* sp. 5 (Fig 4E) exhibited over 99% similarity to Scelionidae sequences from India and Pakistan, respectively (Table 3). *Idris* sp. 3 (Fig 4C) showed 92.6% similarity to *Idris hirsutus* Sunita & Rajmohana, while *Idris* sp. 4 (Fig 4D) displayed 89.8% similarity to a Scelionidae specimen from South Africa (Table 3). The ML tree (Fig 3) revealed each of these *Idris* species as an independent lineage, with no evidence of grouping or formation of a distinct clade.

**Table 3:** BLAST search results for the five species of *Idris.*

Species	NCBI search				BOLD search			
	Accession no.	Identity (%)	Species	Location	BOLD ID	Identity (%)	Species	Location
*Idris* sp. 1	MG351152	89.7	Scelioninae sp.	Canada	GMPJA9337-21	100	Scelionidae sp.	Pakistan
*Idris* sp. 2	MN520969	99.3	Platygastridae sp.	India	GBMNC46287-20	99.3	Scelionidae sp.	India
*Idris* sp. 3	OR699986	92.6	*Idris hirsutus* Sunita & Rajmohana	India	Private	93.6	Scelionidae sp.	Nil
*Idris* sp. 4	KM996909	88.5	*Idris* sp.	USA	SAFSA13139-23	89.8	Scelionidae sp.	South Africa
*Idris* sp. 5	KY842170	99.82	Scelioninae sp.	Pakistan	Private	99.82	Scelionidae sp.	Nil

### 3.2. Host-parasitoid associations

**Fig 5:**
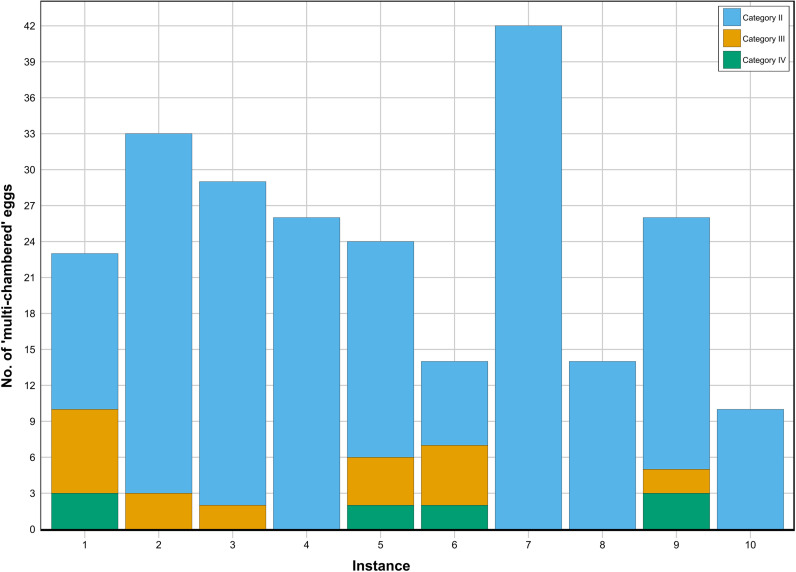
‘Multi-chambered’ spider eggs across different categories, shown instance-wise.

Of the ten egg sacs containing multiple parasitoid progenies, emergence of the same *Idris* species was grouped and treated as single cases, resulting in the following five distinct cases A to E (Table 1). The number of the parasitized ‘multi-chambered’ eggs under three categories (ii, iii, iv) of each instance is depicted in Fig 5. The diameter of the ‘multi-chambered’ eggs of category (ii), (iv) and solitary eggs of category (iv) along with the respective body size of *Idris* are listed in Table 4.

**Table 4:** Morphometric data of parasitized eggs and associated *Idris* species.

Parasitoid	Parasitoids within a single host egg	Size of the parasitoid (mm)	Diameter of the parasitized host egg (mm)
***Idris* sp. 1**	5–6	0.65–0.92	0.8–1.0
***Idris* sp. 2**	1 (solitary)	0.91–1.03	0.8–1.1
	4	0.93–1.03	
***Idris* sp. 3**	4–5	0.91–0.92	0.8–1.0
***Idris* sp. 4**	10	0.85–0.90	1.3–1.9
***Idris* sp. 5**	5	0.88–0.94	0.8–1.0

**Fig 6:**
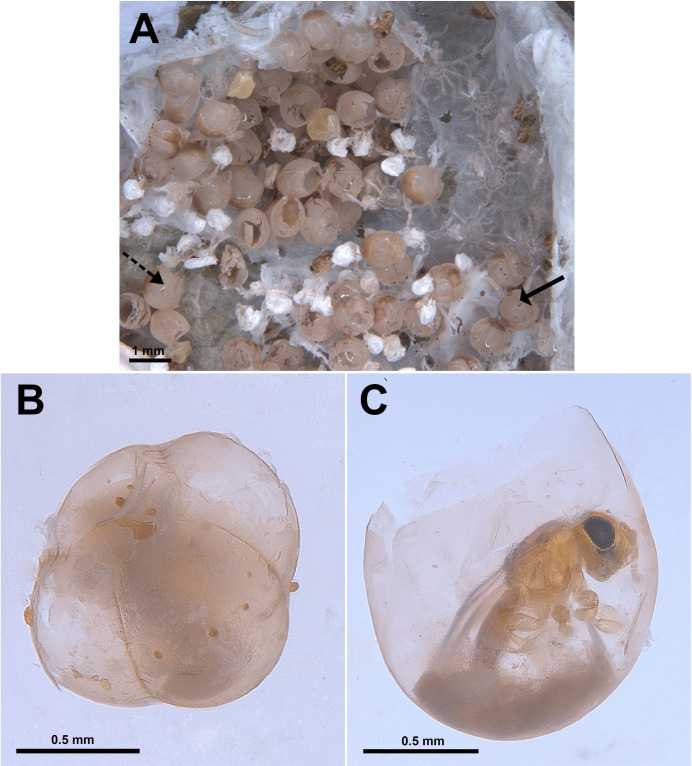
Eggs in egg sac of case B. A. Egg sac with both simple and ‘multi-chambered’ egg (solid arrow = ‘multi-chambered’ eggs, dotted arrow = simple egg); B. Four ‘chambered’ egg under category (ii); C. Simple egg with solitary development of *Idris* sp. 2.

Of these, case B was unique for several reasons:

a) it contained multiple parasitoid genera, both *Idris* sp. and *Odontacolus markadicus* Veenakumari (Fig 4F);
b) all gregarious cases belonged to *Idris* sp. 2 (Fig 4B), both in category (iii) and (iv) and a few were solitary, as evidenced by three eggs without any chambered appearance, each containing single, unemerged parasitoid (Fig 6);
c) all *Idris* sp. 2, whether both solitary and gregarious, were male.

In category (iv), eggs hosting solitary parasitoids were situated towards the interior of the egg sac, while those with multiple progenies were found at the periphery. A total of 72 eggs exhibited solitary parasitoid development, all of which were uniform in size. However, *O. markadicus* was considerably larger in size, at least twice as large as the emerged *Idris* sp. 2 (Fig 4).

In case D, upon dissection of the egg sac, several specimens of *Idris* sp. 4 (Fig 4D) were found entangled within the egg sac. Both in cases D and E, only a few parasitoids emerged in the laboratory during rearing, empty eggs of category (ii) indicated that most of the parasitoids emerged in the field prior to the collection of the spider egg sacs.

## 4. Discussion

A few parasitoid families of Hymenoptera like Scelionidae (Platygastroidea), Encyrtidae, Eulophidae, Eurytomidae, Eupelmidae, Pteromalidae (Chalcidoidea) and Ichneumonidae (Ichneumonoidea) are known to attack the different developmental stages of spiders [4]. *Idris* is solitary idiobiont endoparasitoids of spider eggs [11,18]. This study reports the first recorded instance of multiple specimens of *Idris* emerging from a single spider egg, representing a case of gregarious parasitism. Previously, the emergence of multiple offspring from a spider egg sac was observed primarily in egg predators or pseudoparasitoids [31], where developing parasitoid larvae feed on spider eggs. They mainly belong to chalcidoid families such as Eurytomidae, Pteromalidae, and Eulophidae, as well as Ichneumonidae, along with a few dipterans and neuropterans [4,5].

Solitary and gregarious development are two alternative life-history strategies in parasitoid Hymenoptera [10]. Solitary development is the ancestral state, with gregarious development a derived trait and has evolved independently at least 43 times in 26 different families of Hymenoptera [32]. In Scelionidae, *Telenomus* Haliday [33,34] and *Idris* (present study) are the only genera reported to exhibit gregarious parasitism*.*

Gregarious progeny can at times arise through self-superparasitism but typically results in offspring of varying ages [9]. In this study, all the unemerged parasitoids (categories iii and iv) were at the same developmental stage, they were nearly fully developed adults but unable to emerge out of the egg. Mortality likely occurred during the final stages of development, possibly due to challenges in emerging from the host egg. Polyembryony, as a developmental mode, it is not documented in any idiobiont egg parasitoids and exclusively observed in koinobiont endoparasitoids, including families such as Platygastridae, Braconidae, Dryinidae, and Encyrtidae [35]. The process is intricately synchronized with the host’s development and growth, requiring significant time for completion [35]. Hence only the concept of ‘multiple egg clutch’ [36] offers a plausible explanation for the observed presence of multiple parasitoid individuals of the same developmental age within spider eggs. The laying of multiple egg clutches is influenced by various biotic and abiotic factors [37]. The number of parasitoids developing within a single host are influenced by factors such as host suitability, host population density, and the offspring sex ratio [38]. However, such causative and contributing factors need further exploration.

Solitary species may occasionally exhibit facultative gregarious development [39]. In Case B with *Idris* sp. 2, two emergence patterns were noted: solitary, with one parasitoid per egg, and gregarious, with four parasitoids emerging from a single egg. Such instances of facultative gregariousness have been documented in several idiobiont egg parasitoids, including *Anaphes flavipes* (Förster) [40,41], *A. listronoti* Huber (Mymaridae) [42], and in genus *Trichogramma* Westwood (Trichogrammatidae) [43].

In at least two instances involving *Idris* sp. 1, both male and female progeny were recorded from a single egg (Fig 1C). Based on the analysis of categories (ii) and (iii), all gregarious species of *Idris* exhibited a female-biased sex ratio, except for *Idris* sp. 2 (Case B, Table 1), which produced only male progeny. Arrhenotoky is the common mode of reproduction in Hymenoptera [44]. *Anaphes flavipes* alternates between laying a fertilized and an unfertilized egg in its host weevil egg [38]. However, all-male progeny can also result from other strategies, such as heterotrophic parasitism, as seen in *Encarsia porteri* (Mercet) (Encyrtidae) [45], where female and male eggs are laid on different hosts [10]. Interestingly, the production of single-sex broods may become more common during the transitional phase from solitary to gregarious development, as it helps propagate a non-siblicidal allele [46]. Once gregarious development is fully established, however, selection is likely to favour a shift towards mixed-sex brood production and the evolution of more balanced sex ratios [47,48].

Spider eggs are spherical in shape [49] and, unlike insect eggs, lack a hard outer shell [50]. Instead, they are encased in two layers: the inner vitelline membrane and the outer chorion [51,52]. As the parasitoid larva develops, it depletes most of the resources within the egg. The chorion then hardens and contracts around the walls of the developing pupal cases, turning translucent and revealing the parasitoid pupae from the outside (Fig 1D). This leads to a ‘multi-chambered’ appearance of the host spider eggs. The process is reminiscent of how the polyembryonic egg-larval parasitoid *Copidosoma* Ratzeburg (Encyrtidae) modifies the appearance of its host lepidopteran larvae, creating a multi-chambered look [53,54].

Idiobiont egg parasitoids develop in a ‘closed system,’ where the success of solitary versus gregarious development remains unaffected by fluctuations in resource availability [39]. Larger eggs are generally thought to provide more food and nutrients to support progeny development [55]. In Case D, the eggs housing 10 individuals of *Idris* sp. 4 were notably larger compared to those of other gregarious species (Table 4). However, egg size does not always correlate with the size of the emerged parasitoids; it can also depend on the quality of the egg [56]. Offspring body size is closely linked to maternal allocation decisions, particularly the number of progenies deposited per host [41]. For *Idris* species, whether solitary or gregarious (Case B), the body size remained largely consistent (0.91–1.03 to 0.93–1.03 mm, respectively) (Table 4). The eggs appear sufficiently resource-rich to support the development of multiple individuals without a trade-off between body size and progeny number. This developmental strategy is evolutionarily adaptive, as it enables an increase in offspring number per host without compromising their size. In most gregarious parasitoids, a positive, linear relationship exists between body size and key fitness traits [41]. Body size is crucial for the overall success of parasitoids, influencing key factors such as longevity, fecundity (both daily and lifetime), and fertility. Female body size, in particular, affects fertility, longevity, egg size, foraging efficiency, and competitiveness with other females [43,57].

The host associations of *Idris* with spider species such as *Bianor angulosus*, *B. albobimaculatus*, *Hyllus semicupreus*, *Harmochirus brachiatus*, and *Neoscona* sp. are novel to science. The documentation of five gregarious species of *Idris*, each associated with different spider hosts across seven localities over a span of two years, provides valuable insights. Given the spatiotemporal factors and the diversity of both parasitoid and host species, we assert that these instances represent robust biological processes in nature that were previously overlooked. Such conclusions are only possible through the collection and rearing of host eggs, as simply collecting parasitoid specimens would not reveal the underlying interactions.

With up to 10 individuals developing per host egg through gregarious development, this reproductive strategy offers significant evolutionary advantages over solitary development. The primary advantage lies in the efficient exploitation of a single host, enabling multiple individuals to develop successfully while utilizing the same host resource [36]. At low host densities, gregarious parasitoids increase their numbers per host egg, while solitary parasitoids experience a population decline [38,57]. The gregarious strategy enables parasitoids to respond flexibly to changing environmental conditions, such as variations in host population densities and host characteristics [57]. Three gregarious species of *Idris* (sp. 1, sp. 2, and sp. 3) were documented from the same locality, Raidighi (Fig 2, Table 1). In such cases of sympatry, adopting a gregarious strategy can increase the likelihood of progeny survival despite competition [58].

Gregariousness is more likely to evolve in species-rich taxa [32]. In scelionids, both *Telenomus* [33] and *Idris* [12] are species-rich, suggesting that such taxa may have more ‘tickets in the evolutionary lottery’ [59], possibly due to greater biological diversity or a longer evolutionary history. The ML tree (Fig 3) did not exhibit distinct clustering of gregarious *Idris* species. This underscores the importance of conducting extensive global sampling of *Idris* species, integrating natural history studies with molecular analyses to achieve higher phylogenetic resolution and deeper insights into their evolutionary relationships. The transition from solitary to gregarious development requires specific adaptations [58]. A reduction in aggressiveness, increased tolerance among individuals, and reduced larval mobility within the host are factors that promote gregariousness [60]. Future studies on the gregarious strategy in *Idris* should also focus on the morphology of larval mandibles, behaviour [60] and reduced mobility [58], which are important in differentiating siblicidal and non-siblicidal larvae. Comparative methods, along with genomic research to identify genes linked to reduced mobility in larvae of gregarious parasitoids [46], will provide valuable insights.

## 5. Conclusion

The merit of this study lies in its field-based approach, which provides evidence of diverse and yet-to-be-discovered trophic interactions in nature. Being a life strategy adapted to varying environmental conditions and host population dynamics, gregarious parasitism holds evolutionary significance. However, studies on host-parasitoid interactions and their biology within *Idris* remain limited, warranting further research to uncover these critical aspects. Genetic profiling using molecular tools like single nucleotide polymorphisms can offer further insights into developmental pathways. Histological investigations and controlled rearing experiments can further elucidate the precise mechanisms and driving forces at work.

## Supporting information

S1 Table Mitochondrial COI sequences used in this study for the phylogenetic analysis of *Idris* and their grouping based on ASAP analysis. (PDF)

S1 Fig ASAP analysis. Star indicates the best species partition with lowest score.
(PDF)
