# Supplementary material for: Weaving a new web: gregarious parasitism in Idris Förster (Hymenoptera: Scelionidae) attacking spider eggs
Source: PLoS One. 2025 Feb 25;20(2):e0319209. doi: 10.1371/journal.pone.0319209 (PMC11856503; doi:10.1371/journal.pone.0319209)
Supplement: S1 Table — (PDF) [file pone.0319209.s001.pdf]

S1 Table: Mitochondrial COI sequences used in this study for the phylogenetic analysis of *Idris* and their grouping based on ASAP analysis.

Sl.	Species	ASAP species	NCBI accession no./BOLD id	Location	Reference
1	*Idris* sp. 1	1	PP417915	India	This study
2	*Idris* sp. 1	1	OR621048	India	This study
3	*Idris* sp. 1	1	OR699990	India	This study
4	*Idris* sp. 1	1	OR699992	India	This study
5	*Idris* sp. 1	1	PP426041	India	This study
6	*Idris* sp. 1	1	OR699988	India	This study
7	Scelionidae sp. (*Idris* sp.)	1	GMPJA9337-21	Pakistan	Unpublished
8	*Idris* sp.	2	KP271246	India	Unpublished
9	*Idris* sp.	3	DQ888392	Australia	[1]
10	*Idris* sp. 4	4	PP081609	India	This study
11	*Idris hirsutus*	5	OR699987	India	[2]
12	*Idris hirsutus*	5	OR699986	India	[2]
13	*Idris* sp. 3	6	OR960564	India	This study
14	*Idris* sp. 2	7	OR960562	India	This study
15	Scelionidae sp. (*Idris* sp.)	7	GBMNC46287-20	India	Unpublished
16	Platygastridae sp. (*Idris* sp.)	7	MN520969	India	Unpublished
17	*Idris curtus*	8	MH794513	Malaysia	[3]
18	*Idris curtus*	8	MH794516	Malaysia	[3]
19	*Idris curtus*	8	MH794514	Malaysia	[3]
20	*Idris curtus*	8	MH794523	Philippines	[3]
21	*Idris curtus*	8	MH794532	Philippines	[3]
22	*Idris curtus*	8	MH794515	Malaysia	[3]
23	*Idris curtus*	8	MH794517	Malaysia	[3]
24	*Idris curtus*	8	MH794518	Malaysia	[3]
25	*Idris* sp.	8	GMMBZ093-17	Malaysia	Unpublished
26	*Idris* sp.	8	GMIBB177-17	Indonesia	Unpublished
27	*Idris* sp.	8	GMIAG518-17	Indonesia	Unpublished
28	*Idris* sp.	8	GMIAJ334-17	Indonesia	Unpublished
29	*Idris* sp.	8	GMIAH1461-18	Indonesia	Unpublished
30	*Idris* sp.	8	GMIAI481-17	Indonesia	Unpublished
31	*Idris* sp.	8	GMIAC364-17	Indonesia	Unpublished
32	*Idris* sp.	8	GMIAK7618-18	Indonesia	Unpublished
33	*Idris* sp.	8	GMIAK7502-18	Indonesia	Unpublished
34	*Idris* sp.	8	GMIAE739-17	Indonesia	Unpublished
35	*Idris balteus*	9	MH794526	Philippines	[3]
36	*Idris curtus*	10	MH794524	Philippines	[3]
37	*Idris badius*	11	MH794519	Malaysia	[3]
38	*Idris badius*	11	MH794531	Philippines	[3]
39	*Idris* sp.	12	MH794525	Philippines	[3]

40	*Idris* sp.	13	GMFRT136-15	USA	Unpublished
41	*Idris* sp.	14	KR370117	Canada	Unpublished
42	*Idris* sp.	14	OPPOG601-17	Canada	Unpublished
43	*Idris* sp.	14	MG381111	canada	Unpublished
44	*Idris* sp.	14	MG376377	Canada	[4]
45	*Idris* sp.	14	KR375574	Canada	Unpublished
46	*Idris* sp.	14	KR796963	Canada	[5]
47	*Idris* sp.	14	OPPCG1465-17	Canada	Unpublished
48	*Idris* sp.	14	KR878080	Canada	[5]
49	*Idris* sp.	14	KR364996	Canada	Unpublished
50	*Idris* sp.	14	MG380766	Canada	Unpublished
51	*Idris* sp.	14	KR372630	Canada	Unpublished
52	*Idris* sp.	14	KR895840	Canada	[5]
53	*Idris* sp.	14	KR891008	Canada	[5]
54	*Idris* sp.	14	KR901816	Canada	[5]
55	*Idris* sp.	14	KR885876	Canada	[5]
56	*Idris* sp.	14	KR899397	Canada	[5]
57	*Idris* sp.	14	KR881315	Canada	[5]
58	*Idris* sp.	14	KR886702	Canada	[5]
59	*Idris* sp.	14	KR929293	Canada	[5]
60	*Idris* sp.	14	KR887211	Canada	[5]
61	*Idris* sp.	14	OPPQE2561-17	Canada	Unpublished
62	*Idris* sp.	14	BARSA234-15	Canada	Unpublished
63	*Idris* sp.	14	KR889626	Canada	[5]
64	*Idris* sp.	14	KR371123	Canada	Unpublished
65	*Idris* sp.	14	KR374425	Canada	Unpublished
66	*Idris* sp.	14	KR368133	Canada	Unpublished
67	*Idris* sp.	14	KR372359	Canada	Unpublished
68	*Idris* sp.	14	KR373094	Canada	Unpublished
69	*Idris* sp.	14	KR365634	Canada	Unpublished
70	*Idris* sp.	14	KR366058	Canada	Unpublished
71	*Idris* sp.	14	KR365223	Canada	Unpublished
72	*Idris* sp.	14	KM562074	Canada	Unpublished
73	*Idris* sp.	14	KR374638	Canada	Unpublished
74	*Idris* sp.	14	KR368460	Canada	Unpublished
75	*Idris* sp.	14	KR368089	Canada	Unpublished
76	*Idris* sp.	14	KR371155	Canada	Unpublished
77	*Idris* sp.	14	KR883817	Canada	[5]
78	*Idris* sp.	14	KM562872	Canada	Unpublished
79	*Idris* sp.	14	KM560839	Canada	Unpublished
80	*Idris* sp.	14	KM557803	Canada	Unpublished
81	*Idris* sp.	14	KM564732	Canada	Unpublished
82	*Idris* sp.	14	SSWBA527-21	Canada	Unpublished

83	*Idris* sp.	14	SSWBA533-21	Canada	Unpublished
84	*Idris* sp.	14	KR877316	Canada	[5]
85	*Idris* sp.	14	KR900248	Canada	[5]
86	*Idris* sp.	14	KR880645	Canada	[5]
87	*Idris* sp.	14	KR880643	Canada	[5]
88	*Idris* sp.	14	MG378664	Canada	Unpublished
89	*Idris* sp.	14	KR888700	Canada	[5]
90	*Idris* sp.	14	KR875128	Canada	[5]
91	*Idris* sp.	14	KR887841	Canada	[5]
92	*Idris* sp.	14	KR873564	Canada	[5]
93	*Idris* sp.	14	KR897789	Canada	[5]
94	*Idris* sp.	14	KR877218	Canada	[5]
95	*Idris* sp.	14	KR880448	Canada	[5]
96	*Idris* sp.	14	KR896819	Canada	[5]
97	*Idris* sp.	14	KR879860	Canada	[5]
98	*Idris* sp.	14	KR881848	Canada	[5]
99	*Idris* sp.	14	KR900193	Canada	[5]
100	*Idris* sp.	14	KR887883	Canada	[5]
101	*Idris* sp.	14	KR886532	Canada	[5]
102	*Idris* sp.	14	KR892742	Canada	[5]
103	*Idris* sp.	14	KR895467	Canada	[5]
104	*Idris* sp.	14	KR896159	Canada	[5]
105	*Idris* sp.	14	KR896325	Canada	[5]
106	*Idris* sp.	14	KR887499	Canada	[5]
107	*Idris* sp.	14	KR901384	Canada	[5]
108	*Idris* sp.	14	KR880873	Canada	[5]
109	*Idris* sp.	14	KR895648	Canada	[5]
110	*Idris* sp.	14	KR899463	Canada	[5]
111	*Idris* sp.	14	KR886546	Canada	[5]
112	*Idris* sp.	14	KR874335	Canada	[5]
113	*Idris* sp.	14	KR892193	Canada	[5]
114	*Idris* sp.	14	KR876582	Canada	[5]
115	*Idris* sp.	14	KR892855	Canada	[5]
116	*Idris* sp.	14	KR897354	Canada	[5]
117	*Idris* sp.	14	KR874839	Canada	[5]
118	*Idris* sp.	14	KR886378	Canada	[5]
119	*Idris* sp.	14	KR886223	Canada	[5]
120	*Idris* sp.	14	KR876451	Canada	[5]
121	*Idris* sp.	14	KR876748	Canada	[5]
122	*Idris* sp.	14	KR895390	Canada	[5]
123	*Idris* sp.	14	KR886636	Canada	[5]
124	*Idris* sp.	14	KR886360	Canada	[5]
125	*Idris* sp.	14	KR879423	Canada	[5]

126	*Idris* sp.	14	KR890325	Canada	[5]
127	*Idris* sp.	14	KR892817	Canada	[5]
128	*Idris* sp.	14	KR879830	Canada	[5]
129	*Idris* sp.	14	KR883244	Canada	[5]
130	*Idris* sp.	14	KR897405	Canada	[5]
131	*Idris* sp.	14	KR885139	Canada	[5]
132	*Idris* sp.	14	KR893541	Canada	[5]
133	*Idris* sp.	14	KR887976	Canada	[5]
134	*Idris* sp.	14	KR887210	Canada	[5]
135	*Idris* sp.	14	KR879726	Canada	[5]
136	*Idris* sp.	14	KR792678	Canada	[5]
137	*Idris* sp.	14	MG514562	Canada	[4]
138	*Idris* sp.	14	KR880300	Canada	[5]
139	*Idris* sp.	14	KR886518	Canada	[5]
140	*Idris* sp.	14	KR896617	Canada	[5]
141	*Idris* sp.	14	KR877995	Canada	[5]
142	*Idris* sp.	14	KR896167	Canada	[5]
143	*Idris* sp.	14	KR893253	Canada	[5]
144	*Idris* sp.	14	KR901933	Canada	[5]
145	*Idris* sp.	14	KR883369	Canada	[5]
146	*Idris* sp.	14	KR889851	Canada	[5]
147	*Idris* sp.	14	KR885394	Canada	[5]
148	*Idris* sp.	14	KR889517	Canada	[5]
149	*Idris* sp.	14	KR878621	Canada	[5]
150	*Idris* sp.	14	KR898421	Canada	[5]
151	*Idris* sp.	14	KR895557	Canada	[5]
152	*Idris* sp.	14	KR898586	Canada	[5]
153	*Idris* sp.	14	KM567577	Canada	Unpublished
154	*Idris* sp.	14	MG374651	Canada	Unpublished
155	*Idris* sp.	14	KR890404	Canada	[5]
156	*Idris* sp.	14	KM555993	Canada	Unpublished
157	*Idris* sp.	14	KR881527	Canada	[5]
158	*Idris* sp.	14	KR878951	Canada	[5]
159	*Idris* sp.	14	KR895770	Canada	[5]
160	*Idris* sp.	14	KR894355	Canada	[5]
161	*Idris* sp.	14	KR899457	Canada	[5]
162	*Idris* sp.	14	KR892581	Canada	[5]
163	*Idris* sp.	14	KR892529	Canada	[5]
164	*Idris* sp.	14	KR877442	Canada	[5]
165	*Idris* sp.	14	KR885741	Canada	[5]
166	*Idris* sp.	14	KR876161	Canada	[5]
167	*Idris* sp.	14	KR876908	Canada	[5]
168	*Idris* sp.	14	MG498976	Canada	[4]

169	*Idris* sp.	14	KR897060	Canada	[5]
170	*Idris* sp.	14	KR809267	Canada	[5]
171	*Idris* sp.	14	KR802533	Canada	[5]
172	*Idris* sp.	14	KR889031	Canada	[5]
173	*Idris* sp.	14	KR368403	Canada	Unpublished
174	*Idris* sp.	14	KR801270	Canada	[5]
175	*Idris* sp.	14	KR801020	Canada	[5]
176	*Idris* sp.	14	KR786454	Canada	[5]
177	*Idris* sp.	14	KR873956	Canada	[5]
178	*Idris* sp.	14	KR879606	Canada	[5]
179	*Idris* sp.	14	KR372469	Canada	Unpublished
180	*Idris* sp.	14	KR370909	Canada	Unpublished
181	*Idris* sp.	14	KR372714	Canada	Unpublished
182	*Idris* sp.	14	KR367173	Canada	Unpublished
183	*Idris* sp.	14	KR369422	Canada	Unpublished
184	*Idris* sp.	14	KR371670	Canada	Unpublished
185	*Idris* sp.	14	KR368330	Canada	Unpublished
186	*Idris* sp.	14	KR368444	Canada	Unpublished
187	*Idris* sp.	14	KR370772	Canada	Unpublished
188	*Idris* sp.	14	KR373875	Canada	Unpublished
189	*Idris* sp.	14	KR373109	Canada	Unpublished
190	*Idris* sp.	14	KR371283	Canada	Unpublished
191	*Idris* sp.	14	KR371283	Canada	Unpublished
192	*Idris* sp.	14	KR367538	Canada	Unpublished
193	*Idris* sp.	14	KR368574	Canada	Unpublished
194	*Idris* sp.	14	KR365121	Canada	Unpublished
195	*Idris* sp.	14	KR369018	Canada	Unpublished
196	*Idris bharati*	15	OR665340	India	[6]
197	*Idris bharati*	15	OR665339	India	[6]
198	Scelioninae sp. (*Idris* sp.)	16	MG351152	Canada	Unpublished
199	*Idris* sp.	17	KU064690	Korea	Unpublished
200	*Idris* sp.	17	KU064689	Korea	Unpublished
201	*Idris* sp.	17	KU064691	Korea	Unpublished
202	*Idris* sp. 5	18	PP574570	India	This study
203	Scelionidae sp. (*Idris* sp.)	18	KY842170	Pakistan	Unpublished
204	*Idris fusciceps*	19	MH794522	Thailand	Unpublished
205	*Idris* sp.	20	MH794527	Philippines	[3]
206	*Idris* sp.	20	MH794528	Philippines	[3]
207	*Idris* sp.	20	MH794529	Philippines	[3]
208	*Idris* sp.	21	MH794520	Malaysia	[3]
209	*Idris* sp.	21	MH794521	Malaysia	[3]
210	*Idris* sp.	22	KF679314	Australia	Unpublished
211	*Idris* sp.	23	NZHYM2670-18	New Zealand	Unpublished

212	*Idris* sp.	23	NZHYM2001-13	New Zealand	Unpublished
213	*Idris* sp.	23	NZHYM2007-13	New Zealand	Unpublished
214	*Idris* sp.	23	GMNZA275-14	New Zealand	Unpublished
215	*Idris* sp.	23	GMNZI741-14	New Zealand	Unpublished
216	*Idris* sp.	23	GMNZJ1090-14	New Zealand	Unpublished
217	*Idris* sp.	23	GMNZS1006-14	New Zealand	Unpublished
218	*Idris* sp.	23	GMNZK880-14	New Zealand	Unpublished
219	*Idris* sp.	23	GMNZM518-14	New Zealand	Unpublished
220	*Idris* sp.	23	GMNZA277-14	New Zealand	Unpublished
221	*Idris* sp.	23	GMNZI842-14	New Zealand	Unpublished
222	*Idris* sp.	23	GMNZX664-18	New Zealand	Unpublished
223	*Idris* sp.	23	GMNZX663-18	New Zealand	Unpublished
224	*Idris* sp.	23	GMNZI843-14	New Zealand	Unpublished
225	*Idris* sp.	23	GMNZI830-14	New Zealand	Unpublished
226	*Idris* sp.	23	NZIND068-22	New Zealand	Unpublished
227	*Idris* sp.	23	GMNZS1079-14	New Zealand	Unpublished
228	*Idris* sp.	23	GMNZV177-14	New Zealand	Unpublished
229	*Idris* sp.	23	GMNZL602-14	New Zealand	Unpublished
230	*Idris* sp.	23	GMNZT160-14	New Zealand	Unpublished
231	*Idris* sp.	23	GMNZS1060-14	New Zealand	Unpublished
232	*Idris* sp.	23	GMNZJ1125-14	New Zealand	Unpublished
233	*Idris* sp.	23	GMNZQ221-14	New Zealand	Unpublished
234	*Idris* sp.	24	DQ888391	Australia	[1]
235	*Idris* sp.	24	KF679301	Australia	Unpublished
236	*Idris* sp.	25	MCCAA1449-12	Australia	Unpublished
237	*Idris* sp.	25	GMAEA7562-22	Australia	Unpublished
238	*Idris* sp.	25	GMAEA7902-22	Australia	Unpublished
239	*Idris* sp.	25	GMAEA8761-22	Australia	Unpublished
240	*Idris* sp.	25	NZIND660-22	New Zealand	Unpublished
241	*Idris* sp.	25	CNBAN639-13	Australia	Unpublished
242	*Idris* sp.	25	GMAEA8115-22	Australia	Unpublished
243	*Idris* sp.	25	GMAEA4170-22	Australia	Unpublished
244	*Idris* sp.	25	GMAEA2650-22	Australia	Unpublished
245	*Idris howardi*	26	GMEGO131-14	Egypt	Unpublished
246	*Idris howardi*	26	KUGLA260-22	Canada	Unpublished
247	*Idris howardi*	26	GMGMV1553-20	Germany	Unpublished
248	*Idris howardi*	26	GMPQW132-19	Pakistan	Unpublished
249	*Idris* sp.	27	KR783452	Canada	[5]
250	*Idris* sp.	27	KR794787	Canada	[5]
251	*Idris* sp.	27	KR790488	Canada	[5]
252	*Idris* sp.	27	KR808297	Canada	[5]
253	*Idris* sp.	27	KM567519	Canada	Unpublished
254	*Idris* sp.	27	KR931352	Canada	[5]

255	*Idris* sp.	27	KM568582	Canada	Unpublished
256	*Idris* sp.	27	MG499732	Canada	[4]
257	*Idris* sp.	27	KR788961	Canada	[5]
258	*Idris* sp.	27	KM557169	Canada	Unpublished
259	*Idris* sp.	27	KM568466	Canada	Unpublished
260	*Idris* sp.	27	KR926010	Canada	[5]
261	*Idris* sp.	27	OPPEG1427-17	Canada	Unpublished
262	*Idris* sp.	27	KM561854	Canada	Unpublished
263	*Idris* sp.	27	KM560556	Canada	Unpublished
264	*Idris* sp.	27	KM569072	Canada	Unpublished
265	*Idris* sp.	28	KF679302	USA	Unpublished
266	*Idris howardi*	29	OPPDS1542-17	Canada	Unpublished
267	*Idris howardi*	29	OPPFQ4037-17	Canada	Unpublished
268	*Idris howardi*	29	GMGSG073-12	USA	Unpublished
269	*Idris howardi*	29	GMGSC027-12	USA	Unpublished
270	*Idris howardi*	29	OPPUE926-17	Canada	Unpublished
271	*Idris howardi*	29	OPPQQ706-17	Canada	Unpublished
272	*Idris howardi*	29	BARSC518-16	Canada	Unpublished
273	*Idris elba*	30	MN135850	USA	[7]
274	*Idris elba*	31	MN135848	Mexico	[7]
275	*Idris elba*	31	MN135849	Mexico	[7]
276	*Idris elba*	31	MN135846	Mexico	[7]
277	*Idris elba*	31	MN135845	Mexico	[7]
278	*Idris elba*	31	MN135847	Mexico	[7]
279	*Idris* sp.	32	ASMII13291-22	Australia	Unpublished
280	*Idris* sp.	32	NZIND090-22	New Zealand	Unpublished
281	*Idris* sp.	33	KF679299	Australia	Unpublished
282	Scelionidae sp. (*Idris* sp.)	34	SAFSA13139-23	South Africa	Unpublished
283	*Idris* sp.	35	PLZAZ686-20	Costa Rica	Unpublished
284	*Idris* sp.	35	PLPCF3221-20	Costa Rica	Unpublished
285	*Idris* sp.	35	PLCDC157-20	Costa Rica	Unpublished
286	*Idris* sp.	35	PLSAD416-20	Costa Rica	Unpublished
287	*Idris* sp.	35	PLAFA428-21	Costa Rica	Unpublished
288	*Idris* sp.	35	PLWCZ355-21	Costa Rica	Unpublished
289	*Idris* sp.	35	PLQBQ444-20	Costa Rica	Unpublished
290	*Idris* sp.	35	PLUAZ1140-20	Costa Rica	Unpublished
291	*Idris* sp.	35	PLNDF1180-20	Costa Rica	Unpublished
292	*Idris* sp.	35	PLCFA559-21	Costa Rica	Unpublished
293	*Idris* sp.	35	PLCFA559-21	Costa Rica	Unpublished
294	*Idris* sp.	35	PLABH3327-19	Costa Rica	Unpublished
295	*Idris* sp.	35	PLUAZ2234-20	Costa Rica	Unpublished
296	*Idris* sp.	35	PLXDF2172-20	Costa Rica	Unpublished
297	*Idris* sp.	35	PLFCW557-20	Costa Rica	Unpublished

298	*Idris* sp.	35	PLTCO151-20	Costa Rica	Unpublished
299	*Idris* sp.	35	PLTBB172-20	Costa Rica	Unpublished
300	*Idris* sp.	35	PLADI262-20	Costa Rica	Unpublished
301	*Idris* sp.	35	PLXDH2081-21	Costa Rica	Unpublished
302	*Idris* sp.	35	PLFDE1525-20	Costa Rica	Unpublished
303	*Idris* sp.	35	PLXDG1836-21	Costa Rica	Unpublished
304	*Idris* sp.	35	PLLAU429-20	Costa Rica	Unpublished
305	*Idris* sp.	35	PLXDF2289-20	Costa Rica	Unpublished
306	*Idris* sp.	35	PLXDJ2236-21	Costa Rica	Unpublished
307	*Idris* sp.	35	PLUAT489-20	Costa Rica	Unpublished
308	*Idris* sp.	35	PLWDD447-21	Costa Rica	Unpublished
309	*Idris* sp.	35	PLXDC4019-20	Costa Rica	Unpublished
310	*Idris* sp.	35	PLXDP1204-21	Costa Rica	Unpublished
311	*Idris* sp.	35	PLEDB883-20	Costa Rica	Unpublished
312	*Idris* sp.	35	PLUAY941-20	Costa Rica	Unpublished
313	*Idris* sp.	35	PLSAL224-20	Costa Rica	Unpublished
314	*Idris* sp.	35	JCCCT1573-16	Costa Rica	Unpublished
315	*Idris* sp.	35	PLLAW1316-20	Costa Rica	Unpublished
316	*Idris* sp.	35	PLXCD1094-20	Costa Rica	Unpublished
317	*Idris* sp.	35	PLFET323-21	Costa Rica	Unpublished
318	*Idris* sp.	35	PLABJ1627-19	Costa Rica	Unpublished
319	*Idris* sp.	35	PLXDH2134-21	Costa Rica	Unpublished
320	*Idris* sp.	35	GMCRJ307-13	Costa Rica	Unpublished
321	*Idris* sp.	35	PLXCP1265-20	Costa Rica	Unpublished
322	*Idris* sp.	35	PLEBI149-19	Costa Rica	Unpublished
323	*Idris* sp.	35	PLECX909-20	Costa Rica	Unpublished
324	*Idris* sp.	35	KM996909	USA	Unpublished
325	*Idris* sp.	35	PLLAJ1067-20	Costa Rica	Unpublished
326	*Idris* sp.	35	PLACH401-20	Costa Rica	Unpublished
327	*Idris* sp.	35	PLAEG1064-21	Costa Rica	Unpublished
328	*Idris* sp.	35	PLKDH164-20	Costa Rica	Unpublished
329	*Idris* sp.	35	PLUAY880-20	Costa Rica	Unpublished
330	*Idris* sp.	35	PLFFB719-21	Costa Rica	Unpublished
331	*Idris* sp.	35	JICCX763-16	Costa Rica	Unpublished
332	*Idris* sp.	35	JCCCC2266-16	Costa Rica	Unpublished
333	*Idris* sp.	35	PLXDH1715-21	Costa Rica	Unpublished
334	*Idris* sp.	35	JCCCH691-16	Costa Rica	Unpublished
335	*Idris* sp.	35	JCCCJ450-16	Costa Rica	Unpublished
336	*Idris* sp.	35	JCCCJ153-16	Costa Rica	Unpublished
337	*Idris* sp.	35	JCCCI436-16	Costa Rica	Unpublished
338	*Idris* sp.	35	JCCCG128-16	Costa Rica	Unpublished
339	*Idris* sp.	35	PLABK2060-19	Costa Rica	Unpublished
340	*Idris* sp.	35	PLXCM157-20	Costa Rica	Unpublished

341	*Idris* sp.	35	PLABC3590-18	Costa Rica	Unpublished
342	*Idris* sp.	35	PLXCG1541-20	Costa Rica	Unpublished
343	*Idris* sp.	35	PLXDI2321-21	Costa Rica	Unpublished
344	*Idris* sp.	35	PLZAY388-20	Costa Rica	Unpublished
345	*Idris* sp.	35	PLUAX697-20	Costa Rica	Unpublished
346	*Idris* sp.	35	PLECJ3173-20	Costa Rica	Unpublished
347	*Idris* sp.	35	PLSAN163-20	Costa Rica	Unpublished
348	*Idris* sp.	35	PLPCR482-20	Costa Rica	Unpublished
349	*Idris* sp.	35	PLXDF2287-20	Costa Rica	Unpublished
350	*Idris* sp.	35	PLABE7699-18	Costa Rica	Unpublished
351	*Idris* sp.	35	PLPCN334-20	Costa Rica	Unpublished
352	*Idris* sp.	35	PLABV681-19	Costa Rica	Unpublished
353	*Idris* sp.	35	PLXCP1262-20	Costa Rica	Unpublished
354	*Idris* sp.	35	PLTDD811-20	Costa Rica	Unpublished
355	*Idris* sp.	35	PLFCI694-20	Costa Rica	Unpublished
356	*Idris* sp.	35	PLLAZ1246-20	Costa Rica	Unpublished
357	*Idris* sp.	35	PLZAM174-20	Costa Rica	Unpublished
358	*Idris* sp.	35	PLXDB2885-20	Costa Rica	Unpublished
359	*Idris* sp.	35	PLAAS743-18	Costa Rica	Unpublished
360	*Idris* sp.	35	PLACL379-20	Costa Rica	Unpublished
361	*Idris* sp.	35	PLAAY2048-18	Costa Rica	Unpublished
362	*Idris* sp.	35	PLAEL538-21	Costa Rica	Unpublished
363	*Idris* sp.	35	PLZAX469-20	Costa Rica	Unpublished
364	*Idris* sp.	35	PLZBV317-20	Costa Rica	Unpublished
365	*Idris* sp.	35	PLVDE3530-20	Costa Rica	Unpublished
366	*Idris* sp.	35	PLXDL2241-21	Costa Rica	Unpublished
367	*Idris* sp.	35	PLSAJ456-20	Costa Rica	Unpublished
368	*Idris* sp.	35	PLXDI2273-21	Costa Rica	Unpublished
369	*Idris* sp.	35	PLLAY2506-20	Costa Rica	Unpublished
370	*Idris* sp.	35	PLIDE278-20	Costa Rica	Unpublished
371	*Idris* sp.	35	PLXDI2396-21	Costa Rica	Unpublished
372	*Idris* sp.	35	JCCCR642-16	Costa Rica	Unpublished
373	*Idris* sp.	35	JCCCH685-16	Costa Rica	Unpublished
374	*Idris* sp.	35	JCCCC1934-16	Costa Rica	Unpublished
375	*Idris* sp.	35	PLABG4096-18	Costa Rica	Unpublished
376	*Idris* sp.	35	PLXDI2397-21	Costa Rica	Unpublished
377	*Idris* sp.	35	PLFEM256-21	Costa Rica	Unpublished
378	*Idris* sp.	35	PLHAJ097-19	Costa Rica	Unpublished
379	*Idris* sp.	35	PLSAM321-20	Costa Rica	Unpublished
380	*Idris* sp.	35	PLKDE391-20	Costa Rica	Unpublished
381	*Idris* sp.	35	PLAAC4903-18	Costa Rica	Unpublished
382	*Idris* sp.	35	PLUAY1003-20	Costa Rica	Unpublished
383	*Idris* sp.	35	PLXDD2587-20	Costa Rica	Unpublished

384	*Idris* sp.	35	PLXDC3319-20	Costa Rica	Unpublished
385	*Idris* sp.	35	PLSBE1573-20	Costa Rica	Unpublished
386	*Idris* sp.	35	PLECZ3432-20	Costa Rica	Unpublished
387	*Idris* sp.	35	PLECY2359-20	Costa Rica	Unpublished
388	*Idris* sp.	35	PLUBK414-20	Costa Rica	Unpublished
389	*Idris* sp.	35	PLXDB2976-20	Costa Rica	Unpublished
390	*Idris* sp.	35	PLXDG2168-21	Costa Rica	Unpublished
391	*Idris* sp.	35	PLECQ1157-20	Costa Rica	Unpublished
392	*Idris* sp.	35	PLXCQ661-20	Costa Rica	Unpublished
393	*Idris* sp.	35	PLXDJ2032-21	Costa Rica	Unpublished
394	*Idris* sp.	35	PLECJ4491-20	Costa Rica	Unpublished
395	*Idris* sp.	35	PLADB821-20	Costa Rica	Unpublished
396	*Idris* sp.	35	PLXDF2639-20	Costa Rica	Unpublished
397	*Idris* sp.	35	PLXDK1851-21	Costa Rica	Unpublished
398	*Idris* sp.	35	PLXDD3860-20	Costa Rica	Unpublished
399	*Idris* sp.	35	PLXDJ2159-21	Costa Rica	Unpublished
400	*Idris* sp.	35	PLEDF3489-20	Costa Rica	Unpublished
401	*Idris* sp.	35	PLXCQ733-20	Costa Rica	Unpublished
402	*Idris* sp.	35	PLRDE370-20	Costa Rica	Unpublished
403	*Idris* sp.	35	PLXDF2181-20	Costa Rica	Unpublished
404	*Idris* sp.	35	PLFBT909-19	Costa Rica	Unpublished
405	*Idris* sp.	35	PLECR928-20	Costa Rica	Unpublished
406	*Idris* sp.	35	PLFAC1430-19	Costa Rica	Unpublished
407	*Idris* sp.	35	PLECK3889-20	Costa Rica	Unpublished
408	*Idris* sp.	35	PLPDB1572-20	Costa Rica	Unpublished
409	*Idris* sp.	35	PLTCU329-20	Costa Rica	Unpublished
410	*Idris* sp.	35	PLABG3307-18	Costa Rica	Unpublished
411	*Idris* sp.	35	PLABF6009-19	Costa Rica	Unpublished
412	*Trissolcus basalis*	36	MK720834	USA	Unpublished

References:

1. Murphy NP, Carey D, Castro LR, Dowton M, Austin AD. Phylogeny of the platygastroid wasps (Hymenoptera) based on sequences from the 18S rRNA, 28S rRNA and cytochrome oxidase I genes: implications for the evolution of the ovipositor system and host relationships. Biological J Linn Soc. 2007;91(4): 653–669.

2. Patra S, Rajmohana K, Debnath R, Sen S, Shabnam A, Dinesh KP. A novel host association of *Idris* Förster (Hymenoptera: Scelionidae) with description of a new species from India. J Nat Hist. 2024;58(1-4): 189–203. https://doi.org/10.1080/00222933.2024.2311436

3. Johnson NF, Chen H, Huber BA. New species of *Idris* Förster (Hymenoptera, Platygastroidea) from southeast Asia, parasitoids of the eggs of pholcid spiders (Araneae, Pholcidae). Zookeys. 2018;811: 65–80. https://doi.org/10.3897/zookeys.811.29725

4. DeWaard JR, Ratnasingham S, Zakharov EV, Borisenko AV, Steinke D, Telfer AC, et al. A reference library for Canadian invertebrates with 1.5 million barcodes, voucher specimens, and DNA samples. Sci Data. 2019;6(1):308. https://doi.org/10.1038/s41597-019-0320-2

5. Hebert PD, Ratnasingham S, Zakharov EV, Telfer AC, Levesque-Beaudin V, Milton MA, et al. Counting animal species with DNA barcodes: Canadian insects. Philos Trans R Soc B. 2016;371(1702):20150333. https://dx.doi.org/10.1098/rstb.2015.0333

6. Debnath R, Rajmohana K, Sen S, Shabnam A, Dinesh KP. On baeine wasps (Hymenoptera: Scelionidae) as egg parasitoids of myrmecomorph spiders (Araneae: Salticidae) from India, along with description of a new species of *Idris* Förster. Zool Anz. 2024;309: 66–74. https://doi.org/10.1016/j.jcz.2024.02.001

7. Lomeli-Flores JR, Rodríguez-Rodríguez SE, Rodríguez-Levya E, González-Hernández H, Gariepy TD, Talamas EJ. Field studies and molecular forensics identify a new association: *Idris elba* Talamas, sp. nov. parasitizes the eggs of *Bagrada hilaris* (Burmeister). J Hymenoptera Res. 2019;73: 125–141. https://doi.org/10.3897/jhr.73.38025
