# Supplementary figures and images for: Weaving a new web: gregarious parasitism in Idris Förster (Hymenoptera: Scelionidae) attacking spider eggs
Source: PLoS One. 2025 Feb 25;20(2):e0319209. doi: 10.1371/journal.pone.0319209 (PMC11856503; doi:10.1371/journal.pone.0319209)

**S2 Figure. ASAP analysis.** Star indicates the best species partition with lowest score.

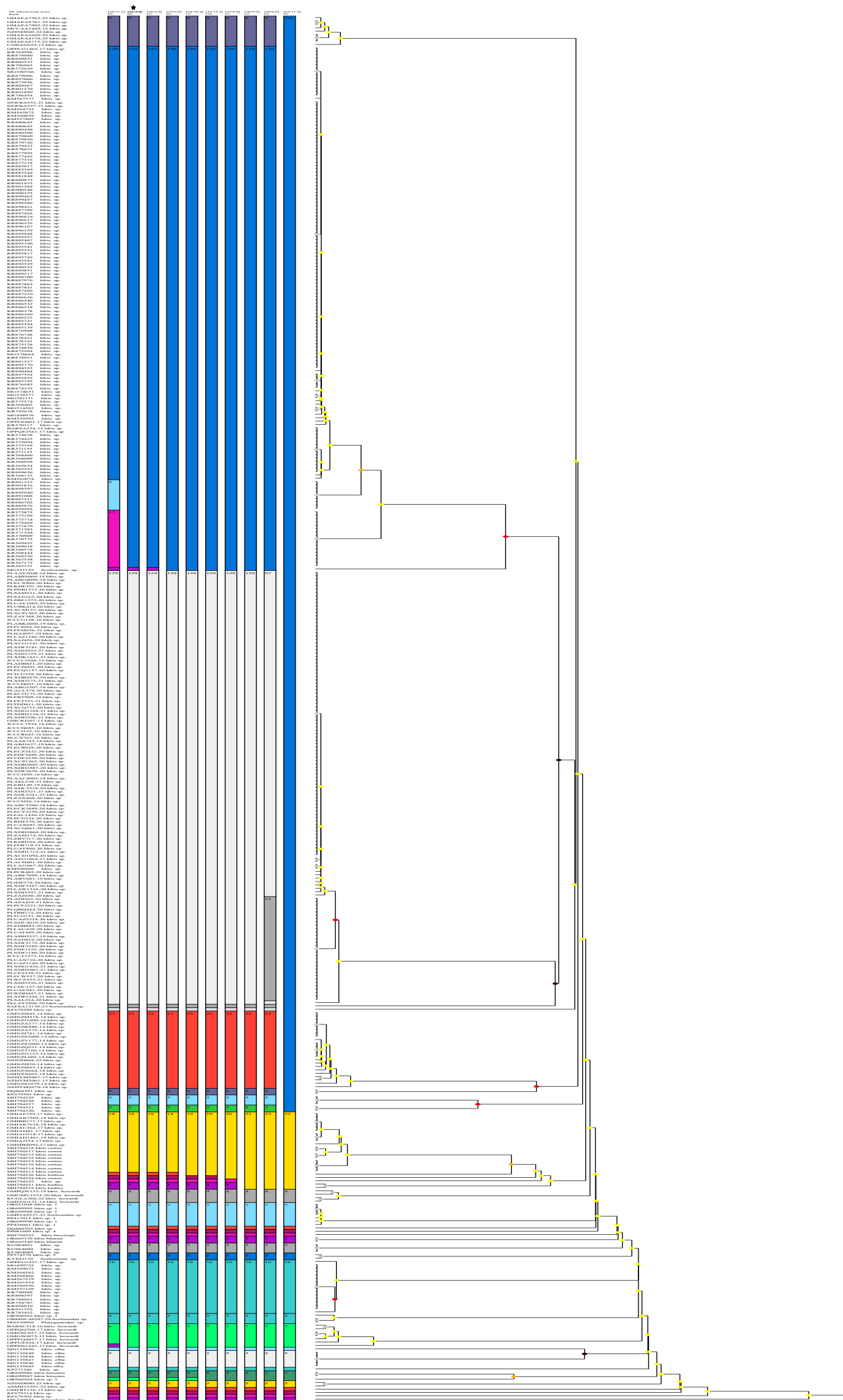

Supplement: S1 Fig — Star indicates the best species partition with lowest score. (PDF) [file pone.0319209.s002.pdf]
